# Vitamin D supplementation for the prevention of total cancer incidence and mortality: An updated systematic review and meta-analysis

**DOI:** 10.1016/j.heliyon.2022.e11290

**Published:** 2022-10-28

**Authors:** Huzaifa Ahmad Cheema, Maurish Fatima, Abia Shahid, Oumnia Bouaddi, Anas Elgenidy, Aqeeb Ur Rehman, Salah Eddine Oussama Kacimi, Mohammad Mehedi Hasan, Ka Yiu Lee

**Affiliations:** a Department of Oncology, King Edward Medical University, Lahore, Pakistan; b Department of Medicine, King Edward Medical University, Lahore, Pakistan; c International School of Public Health, Mohammed VI University of Health Sciences, Casablanca, Morocco; d Faculty of Medicine, Cairo University, Cairo, Egypt; e Faculty of Medicine, Abou-Bekr Belkaid University of Tlemcen, Tlemcen, Algeria; f Department of Biochemistry and Molecular Biology, Faculty of Life Science, Mawlana Bhashani Science and Technology University, Tangail, Bangladesh; g Swedish Winter Sports Research Centre, Department of Health Sciences, Mid Sweden University, Östersund, Sweden

**Keywords:** Meta-analysis, Vitamin D supplements, Cancer incidence, Cancer mortality

## Abstract

**Introduction:**

Previous randomized controlled trials (RCTs) and meta-analyses of RCTs evaluating vitamin D supplementation for the prevention of cancer incidence and mortality have found inconsistent results and no meta-analysis has assessed the quality of the evidence available. We, therefore, aimed to perform an updated meta-analysis by including recent large-scale RCTs and assessing the quality of the pooled evidence.

**Methods:**

We searched several databases and trial registers from inception to April 2022. We used a random-effects model to estimate pooled risk ratios (RRs) and 95% confidence intervals (CIs). We used the Grades of Recommendation, Assessment, Development, and Evaluation (GRADE) considerations to evaluate the certainty of evidence.

**Results:**

We included 13 RCTs in our study. Vitamin D supplementation had no effect on the risk of total cancer incidence (RR 0.99, 95% CI: 0.94–1.04; *I*^*2*^*=* 0%), total cancer mortality (RR 0.93, 95% CI: 0.84–1.03; *I*^*2*^*=* 24%) and total mortality (RR 0.92, 95% CI: 0.82–1.04; *I*^*2*^*=* 36%). The overall quality of evidence was high for all outcomes.

**Discussion:**

Vitamin D supplementation is ineffective in reducing total cancer incidence and mortality in largely vitamin D-replete older adult populations. Future research should be based on populations with a higher prevalence of vitamin D deficiency and should involve more extended follow-up periods.

**Study protocol:**

PROSPERO database, CRD42021285401.

## Highlights

• We assessed the effect of vitamin D supplementation on total cancer incidence and mortality.
• We included 13 randomized controlled trials (RCTs) with 109,543 participants.
• Vitamin D is ineffective in reducing total cancer incidence and mortality in largely vitamin D-replete older adults.
• Future RCTs should focus on populations with a higher prevalence of vitamin D deficiency.

## 1 Introduction

The effects of 25-hydroxyvitamin-D [25(OH)D], also known as calcitriol, have been previously investigated and several roles have been attributed to it. 25(OH)D has been found to affect cell differentiation and apoptosis, and inhibit angiogenesis and the proliferation of cancer cells [1]. Several studies have suggested a negative association between 25(OH)D serum levels and all-cause, cardiovascular and cancer mortality [2, 3, 4]. However, reverse causality could be a potential explanation for these observations.

A previous meta-analysis of cohort studies suggested that higher serum levels of 25(OH)D are associated with lower cancer incidence and cancer mortality [4]. Additionally, higher survival rates were reported among patients with higher levels of circulating 25(OH)D [5, 6, 7]. A previous meta-analysis of RCTs published in 2014 suggested a positive impact of vitamin D supplementation on cancer mortality but no effect on cancer incidence [8]. However, no evidence to support these findings was found in another meta-analysis published in 2018 [9]. The most recent meta-analysis of RCTs published in 2019 suggested that vitamin D supplementation significantly reduced cancer mortality but had no effect on cancer incidence [10]. However, it is worth noting that the beneficial effect seen on mortality was largely influenced by the results of the US VITAL Trial [11] which is contrary to the results of the D-Health Trial [12], a large population-based trial which has been recently published. Therefore, the role of vitamin D in cancer remains to be established. Moreover, no prior meta-analysis has assessed the quality of the existing evidence base.

Several RCTs, including the D-Health Trial, have been published after the most recently published meta-analysis. Therefore, this meta-analysis aims to update the literature and address the inconsistencies regarding the role of vitamin D supplementation compared with placebo in the prevention of the onset of cancer and cancer mortality.

## 2 Materials and methods

Our meta-analysis conforms to the guidelines presented in the Preferred Reporting Items for Systematic Reviews and Meta-Analysis (PRISMA) statement and the *Cochrane Handbook for Systematic Reviews of Interventions* (Supplementary Table 1) [13, 14]. Our protocol has been registered with The International Prospective Register of Systematic Reviews, PROSPERO (CRD42022308968).

### 2.1 Data sources and searches

We searched the Cochrane Central Register of Controlled Trials (CENTRAL, via The Cochrane Library), MEDLINE (via PubMed), Embase (via Ovid), and ClinicalTrials.gov. from inception till April 2022 for any RCTs that involved a comparison of vitamin D at any dose with a control group. We used ProQuest Dissertations and Theses Global (PQDT) and OpenGrey to identify relevant grey literature. Bibliographies of previous meta-analyses and included trials were also screened to identify further RCTs related to our topic. No filter was applied in terms of language or publication period of the included studies.

The complete search strategy for each database can be found in Supplementary Table 2.

### 2.2 Eligibility criteria

We included all RCTs, regardless of the study population, that evaluated the effect of vitamin D supplementation (administered as ergocalciferol or cholecalciferol, regardless of whether in combination with other nutrients such as calcium or not) compared with placebo on total cancer incidence or mortality. Study designs other than RCTs such as observational studies were excluded. We also excluded those RCTs in which the total number of outcome events was ≤10 or the length of follow-up was ≤1 year.

### 2.3 Study selection and data abstraction

All identified records were imported to Mendeley Desktop 1.19.8 (Mendeley Ltd., Amsterdam, The Netherlands) where two authors (AS and MF) working independently screened them, first on the basis of titles and abstracts and then on the basis of full texts in accordance with the predefined eligibility criteria. Any disagreements were resolved by a third author (HAC).

Data from included studies were extracted by two authors (AUR and MMH) into a pre-piloted Excel sheet. A third author (HAC) rechecked the completed extraction sheet and resolved any discrepancies and ensured the accuracy of the data. The following data items were extracted: study characteristics including the year of publication, study location, and follow-up duration, population characteristics such as age and gender, interventions (including co-interventions, types, dosages, and duration of interventions), comparators, and outcomes (primary and secondary outcomes).

### 2.4 Outcome measures

The primary outcomes were total cancer incidence and mortality. The additional outcome was all-cause mortality.

### 2.5 Risk of bias assessment

The methodological quality of our included studies was assessed by two authors (OB and AS) working independently using the revised Cochrane Risk of Bias Tool for randomized controlled trials (RoB 2.0) [15]. The authors assessed bias across five domains: (1) randomisation process; (2 deviations from intended interventions; (3) missing outcome data; (4) measurement of the outcome; and (5) selection of the reported result.

### 2.6 Data synthesis

Statistical analysis was performed using Review Manager (RevMan, Version 5.4; The Cochrane Collaboration, Copenhagen, Denmark). DerSimonian and Laird random-effects models were used to perform meta-analyses. Outcomes were reported as relative risk (RR) along with 95% confidence intervals. The Chi^2^ test and I^2^ statistic were calculated for each group or subgroup analysis to evaluate and quantify the heterogeneity present. I^2^ values were interpreted according to the *Cochrane Handbook for Systematic Reviews of Interventions.* [13] The Chi^2^ test was deemed statistically significant when P < 0.10 [13].

Funnel plots were constructed to assess publication bias and Egger's test was used to check funnel plot asymmetry using Jamovi (version 1.8) [16]. A *P*-value less than 0.10 indicated publication bias.

### 2.7 Subgroup and sensitivity analyses

We conducted subgroup analyses, on our primary outcomes, on the basis of the regimen of vitamin D supplementation, baseline and attained circulating 25(OH)D levels, and the different population groups (postmenopausal women versus general population versus diseased population). For the test for subgroup differences, *P* < 0.05 was deemed statistically significant.

For the primary outcomes, we also conducted a sensitivity analysis by excluding trials that tested the combination of vitamin D and calcium against a placebo.

### 2.8 Certainty of evidence assessment

Two authors (AS and HAC) separately evaluated the certainty of the evidence by using the Grades of Recommendation, Assessment, Development, and Evaluation (GRADE) tool which is based on five domains (risk of bias in individual studies, heterogeneity, imprecision, indirectness, and publication bias) [17, 18]. We judged the pooled effects as imprecise if the optimal information size (OIS) criterion was not met, or the associated 95% CIs included the null effect as well as appreciable benefit or harm [19]. The GRADE approach rates the quality of evidence as one of the four grades: high (the true effect lies close to that of the estimate of the effect), moderate (the true effect is likely to be close to the estimate of the effect, but there is a possibility that it is substantially different), low (true effect may be substantially different from the estimate of the effect), and very low (the true effect is likely to be substantially different from the estimate of effect).

## 3 Results

### 3.1 Study selection and characteristics

**Figure 1:**
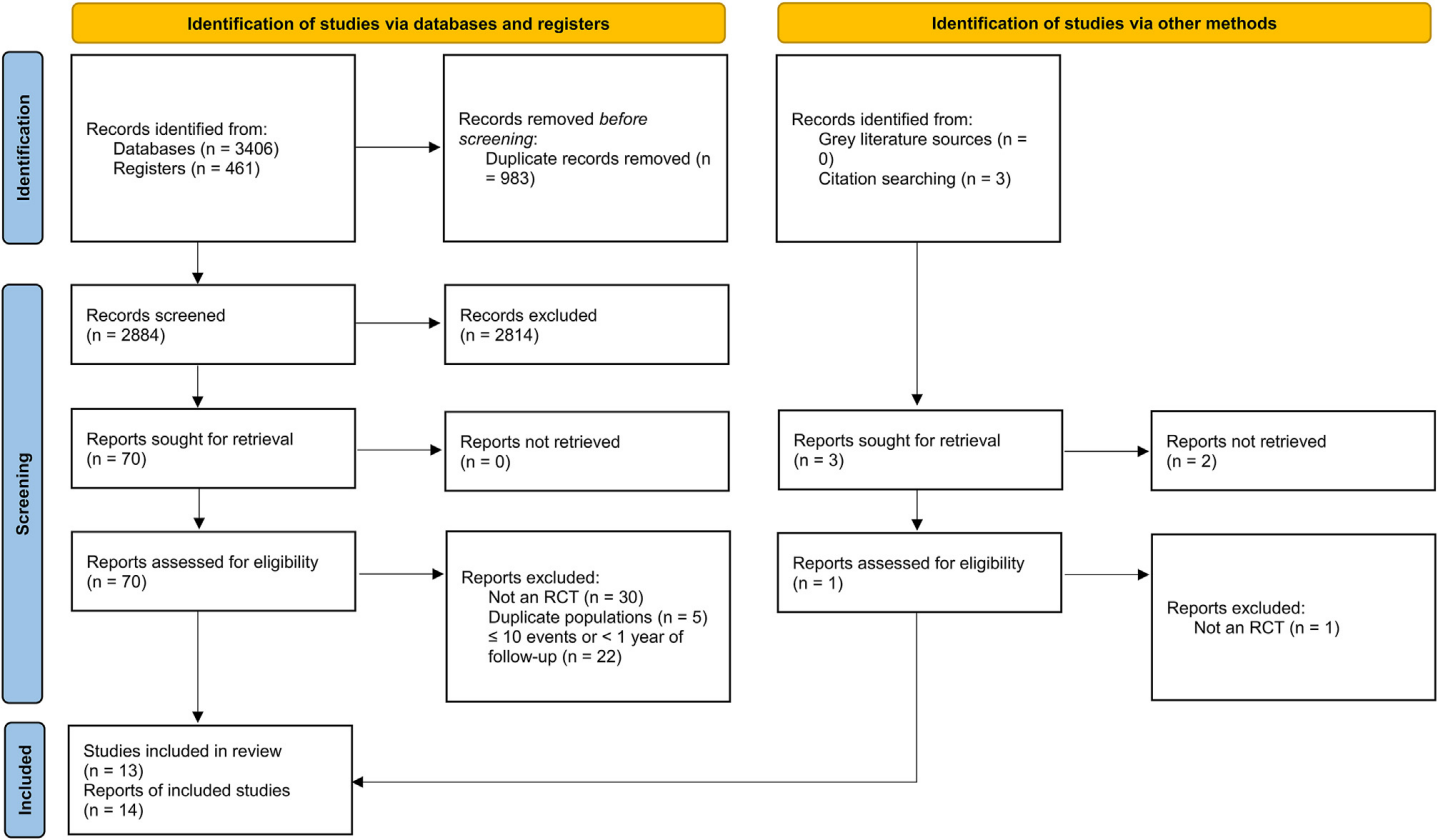
PRISMA 2020 flow chart. Flow chart of included and excluded trials. PRISMA, Preferred Reporting Items for Systematic Reviews and Meta-Analyses.

A comprehensive database search yielded a total of 3867 records while 3 articles were retrieved via citation searching. After removing duplicates (n = 983), the remaining articles were subjected to screening, leaving behind 14 reports of 13 RCTs, comprising 109,543 participants and spanning from 2003 to 2022, that were deemed eligible to be included in this meta-analysis (Figure 1) [11, 12, 20, 21, 22, 23, 24, 25, 26, 27, 28, 29, 30]. Among the included trials, six RCTs were conducted in the USA, two in the UK, two in Australia, one in Finland, one in New Zealand, and one in Norway. In terms of the regimen of Vitamin D intake, eight trials provided Vitamin D3 (400–2000 IU/day) to the participants daily [11, 20, 23, 25, 26, 28, 29, 30], whereas five trials provided an intermittent large dose of Vitamin D3 to the participants (20 000 IU/week to 500 000 IU/year) [12, 21, 22, 24, 27]. At baseline, circulating levels of 25(OH)D in the included trials ranged between 38 and 83 nmol/l, with the intervention group's range reaching between 54 and 135 nmol/l at some point during the follow-up. The post-intervention follow-up duration was approximately 3–10 years. The detailed characteristics of the studies are presented in Table 1.

**Table 1:** Characteristics of included RCTs.

Author	Location	Study population; Mean age	Gender distribution (Male %)	Primary endpoint of trial; Follow-up period	Experimental group (Intervention)	Control group	Baseline Vitamin D change (nmol/L)^a^ (Baseline-- > Follow-up)	Inclusion/Exclusion criteria regarding Vitamin D supplementation
Trivedi [24] 2003	UK	General population; 75 years	76%	Fracture, Total mortality; 5 years	Vitamin D3 where Vitamin. D3: 100,000 IU/4 m (∼833 IU/d)	Placebo	Intervention: NA--> 74 at 4y Control: NA--> 53 at 4y	Excluded current users of Vitamin D supplement
Wactawski-Wende [29] 2006 LaCroix [40] 2009	USA	Postmenopausal women; 62 years	0%	Hip fracture; 7 years (up to 9.7 years)	Vitamin D3+Ca where Vitamin. D3: 400 IU/d and Ca (carbonate): 1000 mg/d	Placebo	Intervention: 42--> 54 at 2 years Control: 42-- > NA	Excluded current users of Vitamin D supplement (≥600 IU/d); Calcitriol
Lappe [28] 2007	USA	Postmenopausal women; 67 years	0%	Fracture; 4 years	Vitamin D3+Ca where Vitamin. D3: 1100 IU/d and Ca (carbonate) 1500 mg/d or (citrate) 1400 mg/d	Calcium	Intervention: 72--> 96 at 1 year Control: 72--> 71 at 1 year	NR
Sanders [21] 2010	Australia	postmenopausal women; 76 years	0%	Fracture; 3–5 years (with 1-year post-intervention follow-up)	Vitamin D3 where Vitamin. D3: 500 000 IU/year (∼1370 IU/d)	Placebo	Intervention: 53--> 58 at trial end Control: 45--> 38 at trial end	Excluded current users of Vitamin D supplement (≥400 IU/d); calcitriol
Avenell [26] 2012	UK	General population; 77 years	15%	Fracture; 2–5.2 years (with 3 years post-intervention follow-up)	Vitamin D3 (w, w/o Ca) where Vitamin D3: 800 IU/d and Ca (carbonate): 1000 mg/d	No Vitamin D3 (w, w/o Ca)	Intervention: 38--> 62 at 1 year Control: 38--> 44 at 1 year	Excluded current users of Vitamin D supplement (200 IU/d); those with past treatment with Vitamin D metabolite or Vitamin D injection
Baron [23] 2015	USA	Individuals with a history of colorectal adenomas; 58 years	63%	Colorectal adenomas; 3.7 years (Range: 3–5 years)	Vitamin D3 (w, w/o Ca) where Vit. D3: 1000 IU/d and Ca (carbonate): 1200 mg/d	No Vitamin D3 (w, w/o Ca)	Intervention: 61--> 81 at trial end Control: 61-- > NA	Limited non-protocol supplement of Vitamin D up to 1000 IU/d
Jorde [22] 2016	Norway	Individuals with impaired fasting glucose and/or impaired glucose tolerance; 62 years	NR	Type 2 diabetes; 5 years	Vitamin D3 where Vitamin D3: 20 000 IU/week (∼2857 IU/d)	Placebo	Intervention: 60--> 122 at trial end Control: 61--> 67 at trial end	Limited non-protocol supplement of Vitamin D (including cod liver oil) up to 400 IU/d
Lappe [25] 2017	USA	Postmenopausal women; 65 years	0%	Total cancer excluding non-melanoma skin cancers; 4 years	Vitamin D3+Ca where Vitamin D3: 2000 IU/d and Ca (carbonate): 1500 mg/d	Placebo	Intervention: 83--> 106 at trial end Control: 82--> 77 at trial end	Limited non-protocol supplement of Vitamin D up to 800 IU/d
Manson [11] 2018	USA	General population including African-Americans by 20%; 67 years	49%	Total invasive cancer, Major cardiovascular events; 5.3 years (Range: 3.8–6.1 years)	Vitamin D3 (w, w/o omega-3 fatty acids) where Vitamin D3: 2000 IU/d and Omega-3 fatty acids: 1 g/d	No vitamin D3 (w, w/o omega-3 fatty acids)	Intervention: 75--> 105 at 1 year Control: 75--> 73 at 1 year	Limited non-protocol supplement of Vitamin D up to 800 IU/d
Scragg [27] 2018	New Zealand	General population; 66 years	58%	CVD; 3.3 years (Range: 2.5–4.2 years)	Vitamin D3 where Vitamin D3: Initial bolus of 200 000 IU followed by 100 000 IU/m (∼3279 IU/d)	Placebo	Intervention: 64--> 120–135 at 0.5–3 years Control: 63--> 70–85 at 0.5–3 years	Excluded current users of Vitamin D supplements including cod liver oil (>600 IU/d if aged 50–70 years; >800 IU/d if aged 71–84 years)
Chatterjee [30] 2021	USA	Overweight and pre-diabetic patients; 60 years	55.50%	Development of cancer 2.9 years (Range: 2–3.5 years)	Vitamin D3 where Vitamin D3: 4000 IU/d	Placebo	Intervention: 69.4-->135.5 at 2 years Control: 70.4-->71.9 at 2 years	Excluded current users of Vitamin D supplements containing vitamin D (1000 IU/d) or Users of medications or conditions that could interfere with the absorption or metabolism of vitamin D
Neale [12] 2022	Australia	General population aged 60–84; 69.3 years	54.10%	Mortality; 5.7 years (Range: 5.4–6.7)	Vitamin D3 where Vitamin D3: 60,000 IU/month	Placebo	Intervention: NA-->115 Control: NA-->77	Excluded current users of Vitamin D supplements >500 IU/d
Virtanen [20] 2022	Finland	General population of males ≥60; Postmenopausal females (≥65); 68.2 years	57%	CVD, Invasive cancer 4.3 years	Vitamin D3 where Vitamin D3: either 1600 IU/d or 3200 IU/d	Placebo	Intervention: 74.9-->120.4 at 1 year Control: 73.7-->72.7 at 1 year	Excluded current users of Vitamin D supplements >800 IU/d

Abbreviations: Ca, calcium; d, day; IU, international unit, M, male; m, month; n, number; NA, not available; Vit, vitamin; w, with; w/o, without.

a To convert to ng/ml, divide by 2.5.

### 3.2 Risk of bias in included studies

Overall, two studies were found to have some concerns of bias, one in the domain of randomization and the other in the domains of randomization and deviations from intended interventions [11, 28], and one study was judged to be at a high risk of bias due to missing outcome data (Supplementary Figure 1) [22]. The remaining studies (76.9%) were found to be at low risk of bias.

### 3.3 Effects of interventions

#### 3.3.1 Primary outcomes

##### 3.3.1.1 Vitamin D supplementation and total cancer incidence

**Figure 2:**
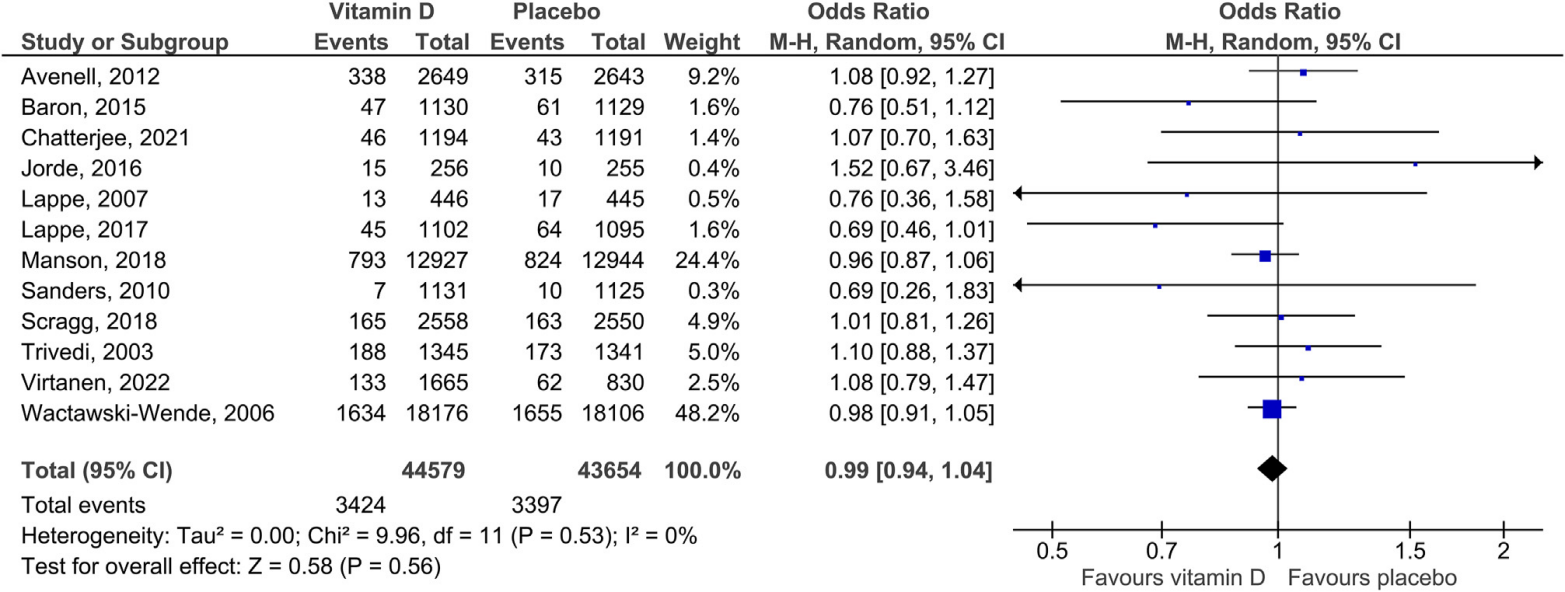
Effect of vitamin D supplementation on total cancer incidence.

Twelve RCTs were included in the analysis of total cancer incidence. The summary RR for intervention versus control group was 0.99 (95% CI: 0.94–1.04, *P* = 0.56). There was no heterogeneity reported (*I*^*2*^ *=* 0%; Figure 2). No funnel plot asymmetry was detected with Egger’s test (*P* = 0.651; Supplementary Figure 2). The overall quality of evidence was rated as high (Supplementary Table 3). There was no statistically significant difference between any of the subgroups: the regimen of vitamin D intake (*P*_*interaction*_ = 0.38), attained 25(OH)D level (*P*_*interaction*_ = 0.64), baseline 25(OH)D level (*P*_*interaction*_ = 0.55), and type of study population (*P*_*interaction*_ = 0.47) (Table 2). The sensitivity analysis reported an RR of 1.00 (95% CI: 0.94–1.07, *P* = 0.94; *I*^*2*^ = 0%) after excluding two trials that reported a combined effect of vitamin D and calcium against a placebo [25, 29].

**Table 2:** Subgroup analysis.

Subgroups	No. of Studies	RR (95% CI)	I^2^ (%)	*P*_*interaction*_ (between subgroups)
**Total Cancer Incidence**				
**By Regimen Intake**				
Daily intake	8	0.98 (0.93–1.03)	0	0.38
Nondaily intake in a large bolus	4	1.05 (0.90–1.23)	0	
**By Attained 25(OH)D**				
>100 nmol/L	6	0.97 (0.90–1.04)	0	0.64
≤100 nmol/L	6	0.99 (0.93–1.06)	0	
**By baseline 25(OH)D**				
>50 nmol/L	9	0.96 (0.89–1.03)	0	0.55
≤50 nmol/L	2	1.00 (0.93–1.08)	20	
**Total Cancer Mortality**				
**By Regimen Intake**				
Daily intake	4	0.87 (0.79–0.97)	0	0.05
Nondaily intake in a large bolus	3	1.06 (0.90–1.25)	8	
**By Attained 25(OH)D**				
>100 nmol/L	4	0.99 (0.80–1.23)	46	0.25
≤100 nmol/L	3	0.88 (0.79–0.98)	0	
**By baseline 25(OH)D**				
>50 nmol/L	5	0.97 (0.82–1.15)	34	0.16
≤50 nmol/L	2	0.88 (0.78–0.99)	0	
**By study population**				
Postmenopausal women	4	0.89 (0.73–1.08)	26	0.47
General population	5	1.01 (0.94–1.08)	0	
Diseased population	3	0.97 (0.70, 1.34)	29	

##### 3.3.1.2 Vitamin D supplementation and total cancer mortality

**Figure 3:**
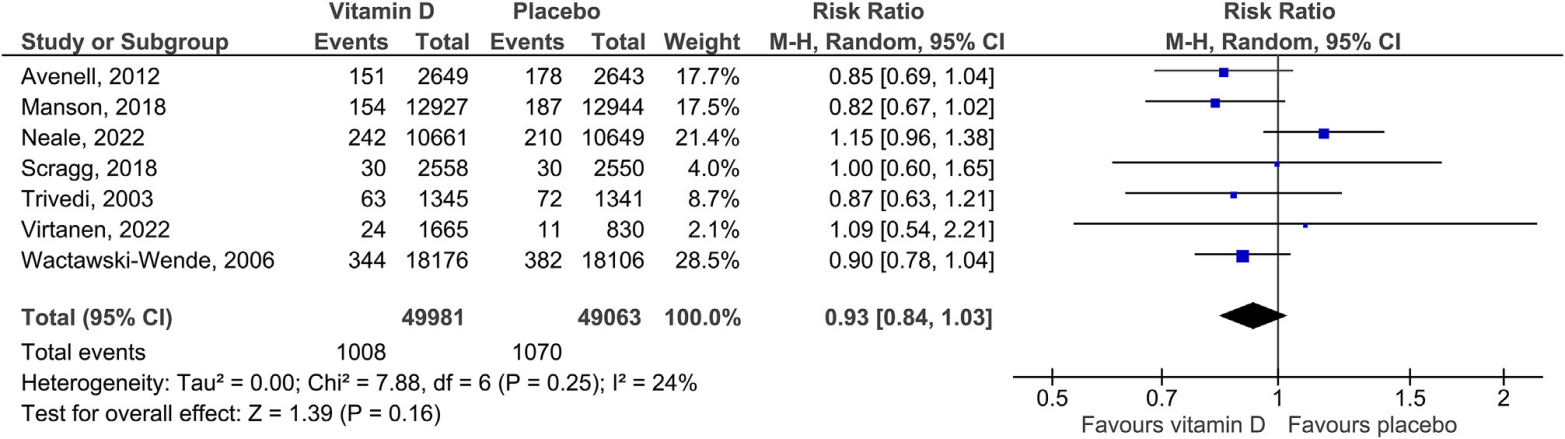
Effect of vitamin D supplementation on total cancer mortality.

We included seven RCTs in the meta-analysis of total cancer mortality. The summary RR for intervention versus control group was 0.93 (95% CI: 0.84–1.03, *P* = 0.16). There was slight heterogeneity reported (*I*^*2*^ = 24%; Figure 3). No funnel plot asymmetry was detected with Egger’s test (*P* = 0.813; Supplementary Figure 3). The certainty of the evidence was rated as high (Supplementary Table 3). The sensitivity analysis reported an RR of 0.94 (95% CI: 0. 82–1.08, P = 0.38; *I*^*2*^ = 34%) after excluding one trial that reported a combined effect of vitamin D and calcium against a placebo [29]. There was no statistically significant difference between the subgroups regarding attained 25(OH)D level (*P*_*interaction*_ = 0.25). Vitamin D decreased the risk of cancer mortality in participants with low baseline 25(OH)D levels (≤50 nmol/L) but not in those with baseline 25(OH)D levels >50 nmol/L; however, the test for subgroup differences was not significant (*P*_*interaction*_ = 0.16; Table 2). When stratified according to the regimen of vitamin D intake, there was a borderline statistically significant difference between the two subgroups (*P*_*interaction*_ = 0.05); daily intake of vitamin D was found to reduce cancer mortality (RR = 0.87; 95% CI: 0.79–0.96, *P* = 0.008, *I*^*2*^ = 0%) but no statistically significant association was observed between infrequent intake of regimen in the form of bolus and cancer mortality (Table 2). The subgroup by type of study population was not conducted due to a lack of data.

#### 3.3.2 Secondary outcome

##### 3.3.2.1 Vitamin D supplementation and total mortality

The summary RR for intervention versus control group was 0.92 (95% CI: 0.82–1.04, *P* = 0.19). There was moderate heterogeneity reported (*I*^*2*^ = 36%; Supplementary Figure 4). No funnel plot asymmetry was detected with Egger’s test (*P* = 0.925; Supplementary Figure 5). The overall certainty of the evidence was rated as high (Supplementary Table 3).

## 4 Discussion

To the best of our knowledge, this is the most comprehensive meta-analysis to date evaluating the effect of vitamin D supplementation on cancer incidence and mortality. We found no significant association between vitamin D supplementation and total cancer incidence, cancer mortality and all-cause mortality. The overall certainty of the evidence was high for all outcomes suggesting that further research is very unlikely to change our confidence in the estimate of effect in largely vitamin D-replete older adult populations. The results also remained consistent across the subgroup analyses. Daily vitamin D intake was found to be associated with reduced cancer mortality compared to bolus intake; however, this observation could be due to chance as the *P*-value for subgroup differences was borderline nonsignificant (*P* = 0.05).

Regarding total cancer incidence, our results are coherent with those reported by recent meta-analyses, which also showed no benefit of vitamin D supplementation in preventing cancer incidence [10, 31]. The evidence from epidemiologic studies regarding the preventive effects of vitamin D based on circulating 25(OH)D levels is limited to the incidence of colorectal and ovarian cancer [32, 33, 34] Anti-neoplastic effects of vitamin D, including anti-proliferation, pro-differentiation, pro-apoptosis, anti-angiogenesis, immune modulation, and miR regulation, provide a possible explanation for these findings [1]. Wei *et al.* reported an inverse association between circulating levels of circulating 25(OH)D and total vitamin D intake with the incidence of colorectal adenoma and recurrent adenomas. However, the analysis did not find any association with supplemental vitamin D indicating that the supplemental sources of vitamin D may not be effective [35]. Meta-analyses of RCTs have also returned no benefit of vitamin D supplementation on colorectal cancer incidence [31, 36]. Results from the D-Health Trial on total and colorectal cancer incidence are forthcoming and may help clarify this further [12].

More importantly, our findings disagree with those reported by recent meta-analyses, which suggested that vitamin D supplementation reduced cancer mortality [10, 31]. However, it is worth pointing out that their pooled estimates were significantly affected by the results of the VITAL trial [11]. Findings from the trials published since then, especially the D-Health Trial, negate these results and our updated meta-analysis also found no beneficial effect of vitamin D on mortality. Indeed, the D-Health Trial found an increased risk of cancer mortality with vitamin D supplementation after excluding the follow-up of the first two years, which was in direct contrast to the VITAL trial [11, 12]. It could be speculated that this disparity might be due to the intermittent dosing regimen used in the D-Health Trial (60 000 IU per month), which might not provide the same benefits as daily dosing regimens [37]. Our meta-analysis also suggested benefits only in trials with daily dosing; however, this needs to be substantiated by trials directly comparing the different dosing regimens.

We also found no reduction in all-cause mortality with vitamin D supplementation, which is congruent with a recent comprehensive meta-analysis [38] but contrary to the findings of Keum *et al.*, which showed a 7% reduction in mortality [10]. Our updated meta-analysis and evidence from large community-based trials indicate that routine vitamin D supplementation is unlikely to confer any mortality benefits in largely vitamin D-replete populations and hence, should be avoided.

The strengths of our study include it being the first meta-analysis to use the GRADE certainty of evidence considerations to inform its conclusions as well as being the largest meta-analysis to date, therefore, having a significantly increased statistical power compared to prior meta-analyses. We also assessed the effects of treatment on cancer mortality and incidence over a period of 3–10 years and excluded studies with few numbers of outcomes to increase the reliability of our results. Moreover, due to the variation in the designs of the studies, we were able to assess several pre-specified subgroups of interest. We did not investigate a large number of subgroups as the rate of type I error increases rapidly with the number of subgroup comparisons [13]. We also used formal statistical tests to compare the subgroups and to inform our conclusions instead of using the different levels of statistical significance within subgroups, which are potentially highly misleading [13], as prior meta-analyses have attempted to do [10, 31]. Our results have little heterogeneity and no evidence of publication bias allowing us to place a high degree of confidence in them.

We would like to acknowledge a few limitations, however. First, there were a limited number of RCTs available and most of the included trials did not have cancer incidence and mortality as primary endpoints. Second, the majority of study participants consisted of Caucasians, and racial differences might modify the intervention effects [39]. Third, we could not assess the intervention effect on organ-specific malignancies as the majority of the trials did not provide site-specific data, and performing subgroup analyses in the absence of a substantial amount of data is not recommended [13]. Moreover, our study was restricted by variable reporting of several important demographic variables such as age and sex by RCTs, precluding any attempt to run a subgroup analysis on them. Lastly, there exists significant heterogeneity in the included RCTs in terms of interventions and inclusion and exclusion criteria of patients. However, we used multiple subgroup and sensitivity analyses to address this heterogeneity.

The null findings of our meta-analysis preclude any recommendations regarding the use of vitamin D supplements as a chemo-preventive measure. Importantly, however, most of the trials had few participants with a 25(OH)D concentration below 50 nmol/L, which is the population that is most likely to benefit. Our subgroup analysis suggested a reduction in cancer mortality in trials with baseline 25(OH)D less than 50 nmol/L; however, the scarcity of studies and a nonsignificant P_interaction_ preclude any firm conclusions. Moreover, longer follow-ups may be required for cancers that progress over decades to demonstrate any benefit. Future large-scale RCTs should be conducted in populations with a higher prevalence of vitamin D deficiency to elicit any potential anticancer benefits of vitamin D supplementation.

In conclusion, vitamin D supplementation is ineffective in reducing total cancer incidence and mortality in largely vitamin D-replete older adult populations. Future research should be based on populations with a higher prevalence of vitamin D deficiency and should involve more extended follow-up periods.

## Declarations

### Author contribution statement

Huzaifa Ahmad Cheema: Conceived and designed the experiments; Performed the experiments; Analyzed and interpreted the data; Contributed reagents, materials, analysis tools or data; Wrote the paper.

Maurish Fatima; Anas Elgenidy: Performed the experiments; Analyzed and interpreted the data; Contributed reagents, materials, analysis tools or data; Wrote the paper.

Abia Shahid: Conceived and designed the experiments; Analyzed and interpreted the data; Contributed reagents, materials, analysis tools or data; Wrote the paper.

Oumnia Bouaddi; Mohammad Mehedi Hasan: Performed the experiments; Contributed reagents, materials, analysis tools or data; Wrote the paper.

Aqeeb Ur Rehman; Salah Eddine Oussama Kacimi: Conceived and designed the experiments; Contributed reagents, materials, analysis tools or data; Wrote the paper.

Ka Yiu Lee, PhD: Contributed reagents, materials, analysis tools or data; Wrote the paper.

### Funding statement

This research did not receive any specific grant from funding agencies in the public, commercial, or not-for-profit sectors.

### Data availability statement

Data will be made available on request.

### Declaration of interest’s statement

The authors declare no conflict of interest.

### Additional information

No additional information is available for this paper.

## Appendix A Supplementary data

Supplementary Material vit D_revised Supplementary Material vit D_revised
